# Psychological Pathway from Obesity-Related Stigma to Anxiety via Internalized Stigma and Self-Esteem among Adolescents in Taiwan

**DOI:** 10.3390/ijerph16224410

**Published:** 2019-11-11

**Authors:** Chung-Ying Lin, Meng-Che Tsai, Chih-Hsiang Liu, Yi-Ching Lin, Yi-Ping Hsieh, Carol Strong

**Affiliations:** 1 Department of Rehabilitation Sciences, The Hong Kong Polytechnic University, Hung Hom, Hong Kong; cylin36933@gmail.com; 2 Department of Pediatrics, National Cheng Kung University Hospital, College of Medicine, National Cheng Kung University, Tainan 700, Taiwan; ache93@yahoo.com.tw; 3 Department of Public Health, National Cheng Kung University Hospital, College of Medicine, National Cheng Kung University, Tainan 700, Taiwan; l72219955612@gmail.com; 4 Department of Early Childhood and Family Education, College of Education, National Taipei University of Education, Taipei 100, Taiwan; ylin11@mail.ntue.edu.tw; 5 Department of Social Work, College of Nursing and Professional Disciplines, University of North Dakota, Grand Forks, ND 58202, USA; yiping66@gmail.com

**Keywords:** overweight, stigma, anxiety, internalized stigma

## Abstract

The objective of this research was to examine the pathway from public stigma, to perceived stigma, to depression in adolescents via internalized stigma. Adolescents in grade 7 through 9 from a junior high school in Changhua County in Taiwan completed self-administered surveys from March to July in 2018. Adolescents were asked questions regarding depressive symptoms, obesity-related perceived stigma, and internalized stigma. Structural equation modeling was used to fit the pathway model. The pathway was first analyzed with the full sample and then stratified by actual and perceived weight status. Our final analytic sample consisted of 464 adolescents. The pathway model suggested an acceptable model fit. Perceived weight stigma (PWS) was significantly associated with internalized stigma regardless of actual or self-perceived weight status. Internalized stigma was significantly associated with anxiety for both actual (β = 0.186) and self-perceived nonoverweight (non-OW) participants (β = 0.170) but not for overweight (OW) participants (neither actual nor self-perceived). For OW adolescents, perceived weight stigma was associated with anxiety. However, the internalization process did not exist. It may be that the influence of perceived weight stigma is larger than internalized stigma on anxiety. It may also be that the level of internalization was not yet high enough to result in anxiety.

## 1. Introduction

Stigma is a fundamental cause of population health inequalities [1]. Indeed, evidence shows that stigmatized status—including, but not limited to, minority sexual orientation, obesity, and mental illness—is the fundamental cause of disparities related to health, health care, and social relationships [1,2,3,4,5]. However, studies that have examined how public stigma is internalized in adolescents attract growing interest among Western countries but is rarely discussed in an Asian context [6,7,8], which may be due to the more conservative cultural environments.

Additionally, weight stigma (or weight bias) among the different minorities seems to be discussed little in the Asian literature [9]. Therefore, there is an existing literature gap for the nature of the relationships between different types of weight stigma and psychological distress among Asian populations, especially in an area with complex cultures such as Taiwan. Specifically, Taiwan has the Chinese culture root with a mindset of Confucianism; however, Taiwan also received Japanese culture from Japan’s colonization, and was later exposed to Westernization [9,10]. Therefore, Taiwan people, especially the youths and adolescents, are highly likely to accept the concept of slim persons as beautiful due to their exposure to Westernization. The youths and adolescents, however, may receive the opinions from their parents or senior relatives to promote the traditional Chinese belief that plumpness is attractive in women and implies wealth for men [11,12].

The evidence of the relationship between weight stigma and psychological distress among Asians was recently published, though it proved insufficient [9,12,13]. Moreover, the association between weight stigma and mental health is supported by a recently published meta-analysis (r = −0.35) [14]. Therefore, we believe that the relationship between weight stigma and psychological distress exists in the Asian population. However, it is unclear how the different types of weight stigma (e.g., weight-related self-stigma, public stigma, and perceived weight stigma) function in the negative consequences on mental health. While perceived stigma indicates the level of stigma an individual perceives, public stigma refers to how a naïve general population endorses biased impressions against a certain minority group that may result in the public doing negative things to this minority group [15]. Once the minority group internalizes the public stigma, they may inflict self-stigma that results in harm to self-esteem [15]. Therefore, using empirical evidence to examine the mechanism of the weight stigma on psychological distress in an Asian population adds knowledge to the current literature.

This study aimed to study the relationships between different types of weight stigma and psychological distress (i.e., anxiety) in subgroups with different objectively defined weight status (i.e., actual overweight (OW) and non-OW groups), and different self-perceived weight status (i.e., perceived OW and non-OW groups), in Taiwan junior high school students. We hypothesized that (1) perceived weight stigma would be a significant predictor for the students’ public stigma on weight, weight-related self-stigma, and anxiety; (2) public stigma on weight would be a significant predictor of the students’ anxiety; and (3) weight-related self-stigma would be a significant predictor of the students’ anxiety. We also hypothesized that the aforementioned hypotheses would be in the same direction between students with different objectively defined weight status and those with different self-perceived weight status.

## 2. Materials and Methods

### 2.1. Participants and Procedures

All students in a junior high school in Chang-Hua County in Taiwan were approached (n = 699) to see if they were interested in participating in the study. Four hundred and sixty-four students with parental consent were asked to fill out a self-reported cross-sectional survey in school (67%). Consenting participants also agreed to have the survey linked to school records for height and weight measured by school nurses. The study protocol was approved by the human research ethics committee at the National Cheng Kung University Hospital (IRB: A-BR-106-009).

### 2.2. Measures

#### 2.2.1. Sociodemographics

Participants completed the information regarding their age, parental education, parental marital status, number of siblings, and whether their parents were immigrants on a background information sheet.

#### 2.2.2. Perceived weight stigma (PWS)

The PWS was assessed using 10 dichotomous items (yes scores = 1 and no scores = 0), which have been used in several previous studies on Chinese populations [4,9,16]. According to the previous literature mentioned above, the 10 PWS items were summed to represent the level of perceived weight stigma. A higher score indicates a higher level of perceived weight stigma. The psychometric properties of the PWS were supported by internal consistency (α = 0.84) [9] and convergent validity (r = 0.35 with Weight Self-Stigma Questionnaire and 0.38 with Weight Bias Internalization Scale) [16].

#### 2.2.3. Weight-related self-stigma

The Weight Bias Internalization Scale (WBIS) was used to assess weight-related self-stigma [17]. The WBIS contains 11 items using a five-point Likert scale that asks participants the agreement on each item statement. A higher WBIS total score indicates a higher level of weight-related self-stigma. The psychometric properties of the WBIS Chinese version were supported by confirmatory factor analysis (comparative fit index [CFI] = 0.991, Tucker–Lewis index [TLI] = 0.989, root mean square error of approximation [RMSEA] = 0.036, and standardized root mean square residual [SRMR] = 0.066) and convergent validity (r = 0.82 with Weight Self-Stigma Questionnaire) [16].

#### 2.2.4. Public stigma on weight

The Beliefs About Obese Persons Scale (BAOP) was used to measure public stigma on weight [18,19]. The BAOP contains eight items using a six-point Likert-type scale (–3 = *strongly disagree* to 3 = *strongly agree*) that asks how an individual believes obesity is under the control of a person with obesity. Six items of the BAOP are negatively worded and they were reverse-coded before summing the eight-item scores. Afterward, the summated score added 24 to examine the belief of an individual toward obesity. A higher score indicates stronger beliefs that people with obesity are unable to control their weight. The psychometric properties of the BAOP Chinese version were supported by confirmatory factor analysis (CFI = 0.958, TLI = 0.941, RMSEA = 0.048, and SRMR = 0.050 [10]).

#### 2.2.5. Anxiety

We used the Hospital Anxiety Depression Scale (HADS), a frequently translated scale that has been validated in Chinese-speaking populations [20], to measure anxiety [21]. The HADS contains 14 items, with 7 items on anxiety and 7 on depression. Because the present study focused on anxiety, only seven items of the HADS were used. Each item was rated using a four-point Likert-type scale scoring from 0 to 3. The negatively worded items in the HADS were reverse-coded and summed up with the other positively worded items. A higher score on the HADS indicates a greater level of anxiety. The psychometric properties of the HADS Chinese version were supported by internal consistency (α = 0.79 to 0.83) and convergent validity (r = 0.57 with depression assessed using HADS) [9].

#### 2.2.6. Actual and perceived weight status

Weight and height were measured at the student health center by a school nurse every year. BMI was calculated and categorized as obese, overweight, normal, or underweight based on their age and gender using criteria published by Taiwan Ministry of Health and Welfare [22]. For perceived weight status, we asked each student in the survey how they viewed their body size on a scale of very thin, slightly thin, just right, slightly obese, and very obese. We merged slightly obese and very obese as the overweight group.

### 2.3. Data Analysis

All the analyses were performed using R software, including the lavaan package for conducting multigroup structural equation modeling (SEM). In the multigroup SEM, we used the same model to conduct twice. The model hypothesized that PWS is associated with BAOP, WBIS, and anxiety; BAOP and WBIS are further associated with anxiety. One multigroup SEM used actual weight status to group the participants into OW or non-OW; another multigroup SEM used self-perceived weight status to group the participants into OW or non-OW. For both SEMs, we applied the same fit indices to examine the data–model fit, where a nonsignificant χ^2^ test together with CFI > 0.9, SRMR < 0.08, and RMSEA < 0.08 indicated satisfactory fit.

## 3. Results

The participant characteristics are presented on Table 1. In brief, no significant differences were found between actual non-OW and actual OW participants in their age (M [SD] = 14.1 [0.8] vs. 14.1 [0.8], respectively; *p* = 0.56), BAOP score (24.6 [3.5] vs. 24.6 [3.4], respectively; *p* = 0.99), and anxiety (13.1 [3.0] vs. 13.2 [2.9], respectively; *p* = 0.64). The actual OW group as compared with the actual non-OW group had higher scores in PWS (11.0 [1.7] vs. 10.4 [1.2], respectively; *p* < 0.001) and WBIS (29.3 [6.2] vs. 24.8 [7.3], respectively; *p* < 0.001). Similar findings were shown for self-perceived weight status groups, except for the significant differences in anxiety: the self-perceived OW group had higher anxiety (13.6 [3.0]) than did the self-perceived non-OW group (12.8 [2.9], *p* = 0.006).

**Figure 1:**
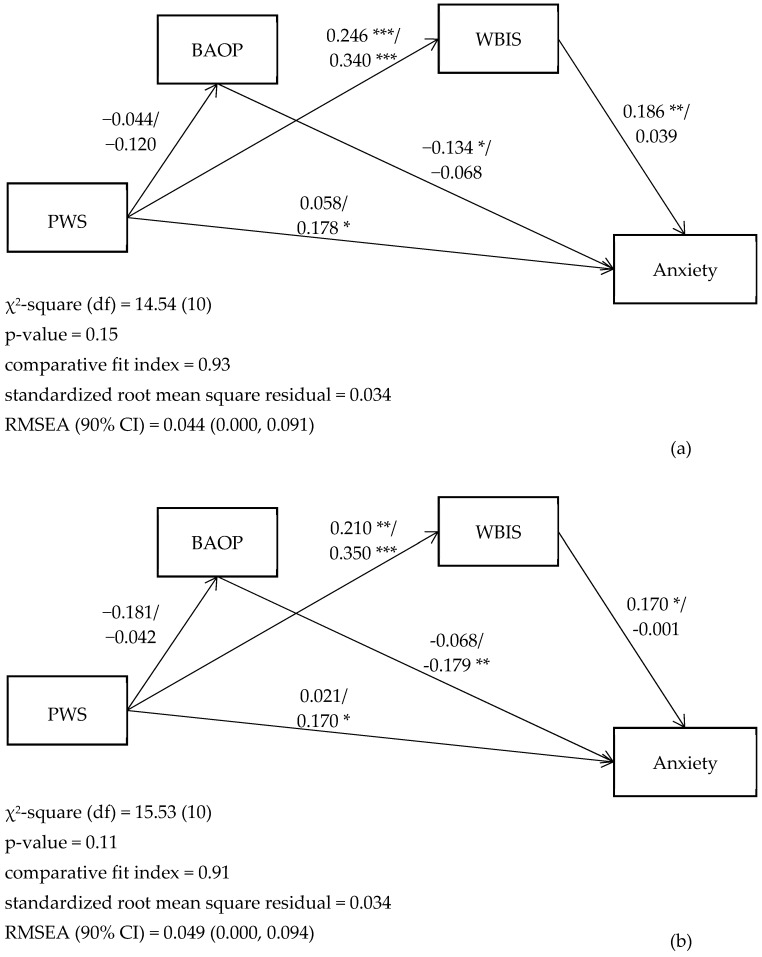
(**a**) Associations between weight bias and anxiety in real nonoverweight/overweight students. (**b**) Associations between weight bias and depression in self-perceived nonoverweight/overweight students. BAOP = belief about obese persons; PWS = perceived weight stigma; WBIS = weight bias internalized scale; RMSEA = root mean square error of approximation. The models are adjusted for age and gender. * *p* < 0.05; ** *p* < 0.01; *** *p* < 0.001.

Our proposed model had satisfactory fit as indicated by the nonsignificant χ2 test (14.54 [10], *p* = 0.15 for SEM with multigroups on actual weight status; 15.53 [10], *p* = 0.11 for SEM with multigroups on self-perceived weight status) together with other fit indices (CFI = 0.93, SRMR = 0.034, and RMSEA = 0.044 for SEM with multigroups on actual weight status; CFI = 0.91, SRMR = 0.034, and RMSEA = 0.049 for SEM with multigroups on self-perceived weight status; Figure 1).

The models further demonstrated that PWS was significantly associated with WBIS regardless of actual or self-perceived weight status (standardized coefficient [β] = 0.246, 0.340, 0.210, and 0.350, for actual non-OW, actual OW, self-perceived non-OW, and self-perceived OW groups, respectively). WBIS was significantly associated with anxiety for both actual (β = 0.186) and self-perceived non-OW participants (β = 0.170) but not for OW participants (neither actual nor self-perceived). PWS was significantly associated with anxiety for both actual (β = 0.178) and self-perceived OW participants (β = 0.170) but not for non-OW participants (neither actual nor self-perceived). Additionally, diverse findings were observed for the relationship between BAOP and anxiety: significant association was found for actual non-OW (β = −0.134) and self-perceived OW participants (β = −0.179), but not for actual OW and self-perceived non-OW participants (Figure 1).

## 4. Discussion

The significant differences between OW and non-OW (regardless of actual or self-perceived) in their perceived weight stigma and weight-related self-stigma are supported by prior studies [9,12]. However, several hypothesized paths were not significant in our results. Specifically, we found no correlation between perceived weight stigma and BAOP score in all the subgroups, which indicates that when an adolescent perceives more weight stigma, the perception will not change their belief in the ability of a person with obesity to control his/her weight. This nonsignificant result may be due to the health education that the adolescents have received. That is, the adolescents have the knowledge of why a person is overweight; therefore, their belief in a person’s ability to control weight is built up and hard to change.

In line with our hypothesis, we found strong associations between perceived weight stigma and weight-related self-stigma among all subgroups. The strong associations support the theory on stigma internalization: a person who is at risk of stigmatization will endorse and accept the biased conception on his/her condition (in our case, OW) after the person is exposed to the unfriendly environment [23,24,25,26]. Consistent with the literature and our hypothesis, perceived weight stigma was associated with anxiety among the actual or perceived OW adolescents [27]. However, perceived weight stigma was not associated with anxiety in actual or perceived non-OW adolescents.

The associations between public stigma on weight and anxiety found in our study contradict our hypothesis. Specifically, our findings indicated that if an actual non-OW adolescent has less public stigma on obesity, the adolescent will have a higher level of anxiety. However, there was no association between the public stigma and anxiety among actual OW adolescents. In terms of the self-perceived weight status, a self-perceived OW adolescent will have a higher level of anxiety if he/she has less public stigma on obesity. However, there was no association between the public stigma and anxiety among self-perceived non-OW adolescents. A possible explanation of the low level of public stigma on weight leading to anxiety is the characteristics of anxiety. People who have anxiety are afraid of being judged [28]. Therefore, people with anxiety characteristics are likely to not judge others, including the weight status. However, our data cannot test our postulation and future studies are thus needed to further investigate this issue.

The association between weight-related self-stigma and anxiety was found in actual or perceived non-OW adolescents. This finding echoes prior research that weight-related self-stigma is a problem beyond people with OW and paying attention to weight-related self-stigma in normal-weight people is necessary [9,29]. However, we found no association between weight-related self-stigma and anxiety in actual or perceived OW adolescents. We suspect that a possible reason is because perceived weight stigma dilutes the association between weight-related self-stigma and anxiety for actual or perceived OW adolescents. Indeed, significant associations were found between perceived weight stigma and anxiety; meanwhile, perceived weight stigma was associated with weight-related self-stigma.

There are some limitations in this study. First, our study design was cross-sectional, which only provides weak evidence in causal effects. That is, the cross-sectional design only determines the associations among our studied variables; however, the temporal associations could not be determined. Although we have prior literature and theory to support the model we proposed, we were unable to ensure the sequence of the different types of weight stigma and anxiety. Therefore, a longitudinal design or a randomized controlled trial to test the causal relationships is needed. Second, all the participants were solely recruited from one Taiwan junior high school through convenience sampling. Hence, our results cannot be generalized to the entire Asian population. For example, both Taiwan and Hong Kong share the same Chinese culture, but studies have suggested the subculture between Taiwan and Hong Kong still needs to be examined [30]. Third, our participants might have included adolescents who had other psychiatric disorders, which could affect stigma and anxiety scores. Thus, future studies might consider assessing the presence of mental illness and conducting a stratified analysis. Fourth, the nature of self-reported questionnaires used in the study was unable to control for social desirability effects. Fifth, students refusing to participate could have been due to stigma related to weight (OW students) and could have potentially introduced selection bias.

Based on our results, future studies may want to explore whether sex is a moderator in our proposed model. Specifically, one study discussed the overlap of obesity- and sex-related issues by examining a sample of college students, in which students were asked to rank the order of pictures of potential sexual partners [31]. When choosing sex partners, people discriminate against obese individuals, particularly for male study participants [31]. Across Western countries, men’s conformity to masculine norms is associated with men’s drive to achieve a certain body image, such as muscularity, leanness, and fitness [32,33]. Among men in sexual minorities, the association between masculine appearance norm violations and body shame is mediated by body surveillance, an act that manifests as self-objectification by constantly surveying one’s body [34]. Such studies in the literature show that although the interactions of sexual orientation, gender-role orientation, and body image have not been examined at the same time and are even sparser in adolescent populations, we may find interplay between these three factors. This is the next step of research in discrimination experiences in adolescents—to examine the interaction between major sources of stigma.

## 5. Conclusions

In conclusion, we found that perceived weight stigma was associated with weight-related self-stigma regardless of weight status; perceived weight stigma was associated with anxiety among OW adolescents; and weight-related self-stigma was associated with anxiety among non-OW adolescents. The aforementioned associations were found consistently in actual and self-perceived weight status. Moreover, eating disturbances were associated with emotional distress regardless of the weight status of our participants. Healthcare providers may want to consider that self-perceived weight status shares the same importance as actual weight of an adolescent when providing services to adolescents coping with their perceived weight stigma and weight-related self-stigma.

## Figures and Tables

**Table 1:** Participant characteristics (N = 464).

	M (SD)		t-Value (*p*)	M (SD)		t-Value (*p*)
	Actual non-OW (N = 289)	Actual OW (N = 175)		Perceived non-OW (N = 248)	Perceived OW (N = 213)	
Age (yr)	14.1 (0.8)	14.1 (0.8)	0.59 (0.56)	14.1 (0.8)	14.2 (0.8)	0.89 (0.37)
Gender (Male) ^a^	133 (46.0%)	98 (56.0%)	4.34 (0.04)	135 (54.4%)	95 (44.6%)	4.43 (0.04)
Height (cm)	159.3 (7.6)	161.0 (8.1)	2.30 (0.02)	159.6 (8.2)	160.4 (7.5)	1.09 (0.28)
Weight (kg)	48.2 (7.3)	69.3 (13.5)	19.10 (<0.001)	48.8 (8.5)	65.0 (14.9)	13.97 (<0.001)
BMI (kg/m^2^)	18.9 (1.9)	26.6 (3.8)	24.76 (<0.001)	19.1 (2.3)	25.1 (4.6)	17.37 ( < 0.001)
PWS	10.4 (1.2)	11.0 (1.7)	4.49 (<0.001)	10.3 (1.3)	11.0 (1.6)	4.79 ( < 0.001)
BAOP	24.6 (3.5)	24.6 (3.4)	0.02 (0.99)	24.5 (3.5)	24.7 (3.5)	0.51 (0.61)
WBIS	24.8 (7.3)	29.3 (6.2)	6.94 (<0.001)	23.9 (7.2)	29.3 (6.0)	8.64 ( < 0.001)
Anxiety ^b^	13.1 (3.0)	13.2 (2.9)	0.47 (0.64)	12.8 (2.9)	13.6 (3.0)	2.77 (0.006)

OW = overweight; BMI = body mass index; PWS = perceived weight stigma; BAOP = belief about obese persons; WBIS = weight bias internalized scale. ^a^ Presented using n (%). ^b^ Assessed using Hospital Anxiety and Depression Scale.
